# Negative impact of internal limiting membrane peeling in vitreous hemorrhage secondary to retinal vein occlusion with macular ischemia

**DOI:** 10.1007/s00417-024-06645-0

**Published:** 2024-10-11

**Authors:** Akihiko Shiraki, Nobuhiko Shiraki, Kazuichi Maruyama, Taku wakabayashi, Susumu Sakimoto, Takatoshi Maeno, Kohji Nishida

**Affiliations:** 1 Department of Ophthalmology, Osaka University Graduate School of Medicine, Rm E7, 2-2 Yamadaoka, Suita, Osaka 565-0871 Japan; 2 Wills Eye Hospital, Mid Atlantic Retina, Thomas Jefferson University, Philadelphia, PA USA; 3 Department of Vision Informatics, Osaka University Graduate School of Medicine, Osaka, Japan; 4 Integrated Frontier Research for Medical Science Division, Institute for Open and Transdisciplinary Research Initiatives, Osaka University, Suita, Japan

**Keywords:** Retinal Vein Occlusion, Vitreous Hemorrhage, Macular Ischemia, Internal Limiting Membrane Peeling

## Abstract

**Purpose:**

To investigate the effect of internal limiting membrane (ILM) peeling on visual outcomes and postoperative epiretinal membrane (ERM) after pars plana vitrectomy (PPV) for vitreous hemorrhage (VH) associated with retinal vein occlusion (RVO) with various degrees of macular ischemia.

**Methods:**

We compared the outcomes of eyes that underwent vitrectomy with and without ILM peeling from 2012 to 2021 with a minimum follow-up of 6 months.

**Results:**

112 charts were analyzed, and 51 eyes met the inclusion criteria. There were 19 eyes with ILM peeling and 32 eyes with non-ILM peeling. Baseline characteristics did not differ significantly. The mean postoperative visual acuity significantly improved at 6 months compared with the mean preoperative visual acuity (*P* < 0.001). Visual improvement was significantly greater in the non-ILM peeling group(*P* < 0.05). Without ischemia within the arcade, there was no significant difference in the visual improvement. In patients with ischemia, the visual improvement in the ILM peeling group was significantly worse than that in the non-ILM peeling group. The incidence of postoperative ERM was significantly higher in the non-ILM peeling; however, there was no significant change in postoperative vision due to the presence of ERM.

**Conclusions:**

Vitrectomy either with or without ILM peeling results in visual improvement in patients with VH associated with RVO; however, it should be uniformly avoiding ILM peeling in cases with pre-existing macular ischemia, as it may significantly lead to a deterioration in visual outcomes.

**Key messages:**

***What is known***

- Pars plana vitrectomy is effective for visual improvement in vitreous hemorrhage associated with retinal vein occlusion.
- The incidence of postoperative epiretinal membrane is variable depending on the surgical approach.

***What is new***

- Avoiding ILM peeling in cases of macular ischemia during vitrectomy is crucial for better visual outcomes.
- Despite higher rates of epiretinal membrane post-surgery in non-ILM peeled eyes, their visual outcomes remain superior to those with ILM peeling.

## Introduction

Retinal vein occlusion (RVO) is the second most common retinal vascular disorder after diabetic retinopathy and is associated with hypertension, smoking, and diabetes [1–4].

RVO is classified into three patterns of ischemia, including central vein occlusion (CRVO), hemi-central retinal vein occlusion (Hemi-CRVO), and branch retinal vein occlusion (BRVO), based on the location of the obstruction [5, 6]. RVO is associated with various complications, such as macular edema, neovascularization, epiretinal membrane (ERM), vitreous hemorrhage (VH), and consequent vision loss. ERM and VH often require surgical intervention. Some papers have reported that ERM removal in RVO results in improved visual acuity [7, 8]. Similarly, vitrectomy for VH can often lead to improved vision, yet it's not uncommon to encounter the development of a postoperative ERM. Reoperation can significantly impede a patient's work and daily life. Even in the absence of any subsequent reduction in visual acuity, the necessity for further surgery can greatly diminish the overall satisfaction with the surgical outcome. Therefore, to avoid the occurrence of postoperative ERM and to prevent any inconvenience to the patient resulting from additional surgery, surgeons may opt to perform an internal limiting membrane (ILM) peeling procedure, which can prevent postoperative ERM [9–12]. However, it can cause inner retinal damage and reduce retinal sensitivity, [13, 14] so its necessity varies by disease. Although avoiding reoperation is crucial, it's preferable to abstain from conducting an ILM peeling procedure to prevent postoperative ERM if it compromises visual prognosis. Especially in RVO, macular ischemia is occasionally seen, and compared to other diseases, the macular region may be more vulnerable, that ILM peeling can potentially yield negative results in RVO cases.

In this study, we examined two groups of patients with and without ILM peeling during surgery for VH associated with RVO. As a result, we report that ILM peeling in patients with macular ischemia is likely to be harmful.

## Materials and methods

This retrospective chart review included a consecutive series of 112 eyes (112 patients) with VH secondary to BRVO or Hemi-CRVO, treated with primary PPV between January 2012 and October 2021 at Osaka University Hospital. Among these patients, eyes that were followed up for at least 6 months after surgery were included. Some eyes were excluded from the analysis for the following reasons: (1) no OCT images during follow-up, (2) prior vitrectomy, (3) presence of tractional retinal detachment and age-related macular degeneration, and (4) CRVO. Institutional Review Board (IRB)/Ethics Committee approval was obtained, and the study adhered to the tenets of the Declaration of Helsinki. The patients provided written informed consent after receiving a detailed description of the surgical procedure.

### Surgical technique

We performed standard vitrectomy using a 25-gauge or 27-gauge system (Constellation Vitrectomy System; Alcon Laboratories, Inc., Fort Worth, TX). Core vitrectomy was performed with triamcinolone acetonide　(MaQaid, Wakamoto Pharmaceutical, Tokyo, Japan) to visualize the vitreous gel and posterior hyaloid. We also performed peripheral vitreous base shaving. After core and peripheral vitreous base shaving for the removal of VH, Endolaser photocoagulation was performed in the areas of peripheral nonperfusion. The need for intraoperative ILM peeling was at the discretion of the surgeon. ILM peeling was performed using triamcinolone acetonide, indocyanine green, or Brilliant Blue G. All phakic cases underwent combined phacoemulsification and intraocular lens implantation.

### Data analysis

We collected the following parameters from medical records and surgical notes: ophthalmic history, age, sex, preoperative BCVA, postoperative BCVA at 1, 3, and 6 months, and the incidence of postoperative ERM, which was defined as grade 1 or higher according to the classification published by Govetto et al. [15] For BCVA worse than count finger(CF), these conversion were used as previously reported. CF was converted to a logMAR value of 2.6, hand motion(HM) to 2.7, light perception(LP) to 2.8 and no light perception(NLP) to 2.9 [16, 17]. Swept-source OCT (DRI-OCT; Topcon Medical Systems, Tokyo, Japan) (PLEX Elite 9000; Carl Zeiss Meditec, Dublin, CA, USA) or SD-OCT (Cirrus HD-OCT, Carl Zeiss Meditec, Dublin, California, USA) (RTVue XR Avanti, Optovue, Inc., Fremont, CA, USA) was performed regularly to evaluate the postoperative ERM for 1 month, 3 months, and 6 months. In addition, two of the authors (AS and NS) evaluated the OCT images to identify the presence of ERM formation, with masking of the surgical notes regarding whether ILM peeling was performed.

### Ischemia within arcade and FAZ ischemia

Ischemia within the arcade was defined as the presence of vascular sheathing within the arcade and evaluated by two surgeons using intraoperative video separately. Ischemia of the foveal avascular zone (FAZ) was evaluated using fluorescein angiography (FA) or optical coherence tomography angiography (OCTA) from preoperative to 12 months postoperative. Patients with preoperative evaluation were only included if FAZ ischemia had already occurred. Cases in which RVO recurred during the course of the disease were excluded.

FA was performed using a confocal scanning laser ophthalmoscope (HRA2; Heidelberg Engineering Inc., Dossenheim, Germany), DX50 retinal camera (Topcon, Tokyo, Japan), or Optomap panoramic 200Tx imaging system (Optos, PLC, Dunfermline, United Kingdom). Alternatively, OCTA was performed using spectral-domain OCT (RTVue XR Avanti; Optovue, Inc., Fremont, CA, USA) or swept-source optical coherence tomography angiography (OCTA) (PLEX Elite 9000; Carl Zeiss Meditec, Dublin, CA). To provide a clear definition of cases with FAZ ischemia, we established specific criteria. These cases were identified by meeting the following conditions: the non-perfusion area was connected to the FAZ, there was a distinct morphological difference between the upper and lower regions of the FAZ, and an asymmetrical enlargement of the avascular zone was observed. Due to the possibility of RVO developing without awareness, we refrained from comparing RVO to the contralateral eye, which may not be truly normal. Instead, we identified cases of FAZ ischemia with vertical asymmetry. To ensure a clear determination of FAZ ischemia, cases of CRVO that lacked vertical asymmetry were excluded from the analysis. This exclusion was implemented to avoid any ambiguity in identifying FAZ ischemia. To determine FAZ ischemia, the two surgeons reviewed the FA or OCTA images.

### Statistical analysis

For statistical analyses, we measured BCVA using the Landolt C acuity chart and analyzed with the logarithm of the minimal angle of resolution (logMAR) scale. Where appropriate, the Mann–Whitney rank-sum test, Student’s t-test, and Fisher exact test were used to compare parameters between the two groups. All statistical analyses were performed using the JMP Pro Software (SAS Inc., Cary, NC, USA). Statistical significance was set at *P* < 0.05. We used univariate regression analysis to investigate the association of the logMAR BCVA and the improvement of BCVA after surgery with several parameters, including age, sex, preoperative logMAR BCVA, ILM peeling technique, and macular ischemia. And we used multiple regression to investigate the BCVA and improvement with above mentioned parameters, excluding confounding factors. All statistical analyses were performed using JMP Pro Software (SAS Inc., Cary, NC, USA). A P-value of < 0.05 was considered statistically significant. A logistic regression model was constructed using the minimum Bayesian information criterion-based forward stepwise selection method with a significance level of 0.05.

## Results

A total of 112 eyes (112 patients) were analyzed and 49 eyes of 49 patients met the criteria for inclusion into the study. Eyes that lacked visual acuity measurements at 1, 3, or 6 months postoperatively were excluded from this study. Patient characteristics are summarized in Table 1. The mean age of the patients was 69.9 ± 9.9 years (range, 47–88 years). The mean preoperative BCVA (logMAR) was 1.89 ± 0.90. In addition, ischemia within the arcade was observed in 38 eyes, and FAZ ischemia in 23 eyes. We categorized the 49 eyes into two groups for analysis. We compared 31 eyes in the non-ILM peeling group and 18 eyes in the ILM peeling group. Baseline characteristics including age, gender, laterality, cataract surgery status, ischemia within the arcade, and FAZ ischemia, did not differ significantly between the two groups.

**Table 1:** Patient Characteristics

	Overall	ILM Peeling	Non-ILM Peeling	P value
No. of eyes/ No. of patients	49/49	18/18	31/31	
Age (years) (mean ± SD; range)	69.6 ± 9.9 (47–88)	68.4 ± 9.0 (52–87)	70.3 ± 10.5 (47–88)	0.37
Gender, No. (%)				
Men	22 (45)	7(38.9)	15 (48.4)	
Women	27 (55)	11 (61.1)	16 (51.6)	0.56
Eye, No. (%)				
Right	25 (51)	8 (44.4)	16 (51.6)	
Left	24 (49)	10 (55.6)	15 (48.4)	0.77
Combined cataract surgery, No. (%)	37 (76)	16 (88.9)	21 (67.7)	0.17
Surgical procedure during PPV, No. (%)				
25-gauge	46 (94)	17 (94.4)	29 (93.5)	
27-gauge	3 (6)	1 (5.6)	2 (6.5)	0.92
Intravitreal tamponade during PPV, No. (%)				
Air	3 (6)	2 (11.1)	1 (3.2)	
None	46 (94)	16 (88.9)	30 (96.8)	0.55
ILM dyeing, No				
TA		12 (66.7)	0	
ICG		6 (33.3)	0	
ischemia within arcade, No. of eyes	38	15	23	0.72
FAZ ischemia, No. of eyes	25	11	14	0.50

*ILM *internal limiting membrane,* SD* standard deviation, *PPV* pars plana vitrectomy, *TA* triamcinolone acetonide, *ICG* indocyanine green, *FAZ* fovea avascular zone

### Visual outcome

We obtained 6-month follow-up data, and visual outcomes are summarized in Table 2. In ILM peeling group, the mean BCVA significantly improved from 1.60 ± 1.06 to 0.20 ± 0.41 at 6 months (*P* < 0.001). In the non-ILM peeling group, the mean BCVA also significantly improved from 2.06 ± 0.76 to 0.004 ± 0.18 at 6 months (*P* < 0.001). There was no significant difference in preoperative BCVA or in postoperative logMAR BCVA at 1 month between the two groups (*P* = 0.17 preoperatively and *P* = 0.28 at 1 month). However, the postoperative logMAR BCVA in non-ILM peeling group was significantly better than in the ILM peeling group (*P* = 0.044 at 3 months and *P* = 0.048 at 6 months). Visual improvement was significantly greater in the non-ILM peeling group at 6 months (*P* = 0.16 at 1 month, *P* = 0.051 at 3 months, and *P* = 0.029 at 6 months). And in ILM peeling group, there is no significant difference caused by staining dye (*P* = 0.4 at 1 month, *P* = 0.83 at 3 months, and *P* = 0.71 at 6 months).

**Table 2:** Visual outcomes after Surgery

Parameter	ILM Peeling	Non-ILM Peeling	P value
Preoperative BCVA			
LogMAR (mean ± SD)	1.60 ± 1.06	2.06 ± 0.76	0.17
Postoperative BCVA at 1 month			
LogMAR (mean ± SD)	0.19 ± 0.39	0.14 ± 0.52	0.28
Postoperative BCVA at 3 months			
LogMAR (mean ± SD)	0.25 ± 0.42	0.04 ± 0.20	0.044
Postoperative BCVA at 6 months			
LogMAR (mean ± SD)	0.20 ± 0.41	0.004 ± 0.18	0.048
Improvement of Postoperative BCVA at 1 month			
LogMAR (mean ± SD)	-1.40 ± 1.07	-1.92 ± 0.77	0.16
Improvement of Postoperative BCVA at 3 months			
LogMAR (mean ± SD)	-1.37 ± 1.09	-2.02 ± 0.70	0.051
Improvement of Postoperative BCVA at 6 months			
LogMAR (mean ± SD)	-1.39 ± 1.06	-2.05 ± 0.72	0.029

*ILM* internal limiting membrane, *BCVA* best-corrected visual acuity, *logMAR* logarithm of the minimum angle of resolution, *SD* standard deviation

### Postoperative ERM and visual acuity

In the ILM peeling group, the postoperative ERM developed in 1 (5.9%) of 17 eyes at 1 month, 1 (5.9%) of 17 at 3 months, and 1 (6.3%) of 16 at 6 months. In the non-ILM peeling group, the postoperative ERM was 14 (50%) of 28 eyes at 1 month, 16 (53.3%) of 30 at 3 months, and 17 (56.7%) of 30 at 6 months. The incidence of postoperative ERM was significantly greater in the non-ILM peeling group (*P* < 0.001). The postoperative BCVA in patients with ERM was 0.20 ± 0.64 at 1 month, 0.10 ± 0.24 at 3 months, and 0.06 ± 0.22 at 6 months. The postoperative BCVA in patients without ERM was 0.13 ± 0.36 at 1 month, 0.13 ± 0.36 at 3 months, and 0.09 ± 0.35 at 6 months. There was no significant difference in the postoperative BCVA between the ERM and non-ERM groups (*P* = 0.73 at 1 month; *P* = 0.99 at 3 months; *P* = 0.82 at 6 months).

Additionally, there was no significant difference in the incidence of ERM between the ischemia group and the non-ischemia group (For Ischemia within arcade, *P* = 0.73 at 1 month, *P* = 0.73 at 3 months, and *P* = 0.49 at 6 months; For FAZ ischemia, *P* = 0.26 at 1 month, *P* = 0.48 at 3 months, and *P* = 0.46 at 6 months).

### Ischemia and postoperative visual acuity

The patients were divided into the presence or absence of ischemia within the arcade; ischemic group and non-ischemia. These two groups were compared by the presence or absence of ILM peeling to examine the factors related to poor visual improvement after ILM peeling. Thirty-eight eyes of 49 with ischemia within the arcade in fundus photograph were analyzed (Table 3).

**Table 3:** Ischemia within arcade and Postoperative Visual Outcomes

Parameter	Overall	ILM Peeling	Non-ILM Peeling	P value
Preoperative BCVA				
LogMAR (mean ± SD)	2.00 ± 0.90	1.64 ± 1.09	2.24 ± 0.67	0.13
Postoperative BCVA at 1 month				
LogMAR (mean ± SD)	0.23 ± 0.51	0.25 ± 0.40	0.22 ± 0.58	0.38
Postoperative BCVA at 3 months				
LogMAR (mean ± SD)	0.17 ± 0.32	0.30 ± 0.42	0.089 ± 0.20	0.08
Postoperative BCVA at 6 months				
LogMAR (mean ± SD)	0.13 ± 0.32	0.26 ± 0.42	0.047 ± 0.19	0.09
Improvement of Postoperative BCVA at 1 month				
LogMAR (mean ± SD)	-1.77 ± 0.95	-1.39 ± 1.11	-2.02 ± 0.75	0.14
Improvement of Postoperative BCVA at 3 months				
LogMAR (mean ± SD)	-1.83 ± 0.94	-1.34 ± 1.13	-2.15 ± 0.63	0.034
Improvement of Postoperative BCVA at 6 months				
LogMAR (mean ± SD)	-1.87 ± 0.93	-1.38 ± 1.09	-2.19 ± 0.66	0.015

*ILM* internal limiting membrane, *BCVA* best-corrected visual acuity, *logMAR* logarithm of the minimum angle of resolution, *SD* standard deviation

In 38 eyes with ischemia within the arcade, the preoperative BCVA was 2.00 ± 0.90 and the postoperative visual acuity was 0.17 ± 0.32 at 3 months (*P* < 0.001) and 0.13 ± 0.32 at 6 months (*P* < 0.001). In the ILM peeling group, the BCVA improved from 1.64 ± 1.09 at baseline to 0.30 ± 0.42 at 3 months(*P* < 0.001) and to 0.26 ± 0.42 at 6 months (*P* < 0.001). The visual improvement was -1.34 ± 1.13 at 3 months and -1.38 ± 1.09 at 6 months. In the non-ILM peeling group, the BCVA improved from 2.24 ± 0.67 at baseline to 0.089 ± 0.20 at 3 months (*P* < 0.001) and to 0.047 ± 0.19 at 6 months (*P* < 0.001). The visual improvement was -2.15 ± 0.63 at 3 months and -2.19 ± 0.66 at 6 months. The visual improvement at 3 and 6 months in the ILM peeling group was significantly worse than that in the non-ILM peeling group (*P* = 0.034, *P* = 0.015).

However, no significant difference was found between the ILM peeling and non-ILM peeling groups in visual improvement from preoperative BCVA at 3 months (*P* = 0.76) and 6 months (*P* = 0.76) in cases without ischemia within the arcade.

Then, the patients were categorized into two groups according to the presence of FAZ ischemia and postoperative BCVA and visual improvement was compared by the presence or absence of ILM peeling to examine the potential factors related to poor visual improvement after ILM peeling. Twenty-three eyes of 36 with ischemia within the arcade in FA or OCTA were analyzed (Table 4).

**Table 4:** Foveal Area Ischemia in Angiography and Visual Outcomes

Parameter	Overall	ILM Peeling	Non-ILM Peeling	P value
Preoperative BCVA				
LogMAR (mean ± SD)	1.83 ± 0.99	1.30 ± 1.18	2.23 ± 0.58	0.07
Postoperative BCVA at 1 month				
LogMAR (mean ± SD)	0.13 ± 0.22	0.13 ± 0.16	0.12 ± 0.26	0.73
Postoperative BCVA at 3 months				
LogMAR (mean ± SD)	0.14 ± 0.23	0.20 ± 0.24	0.09 ± 0.23	0.18
Postoperative BCVA at 6 months				
LogMAR (mean ± SD)	0.10 ± 0.23	0.16 ± 0.26	0.04 ± 0.20	0.25
Improvement of Postoperative BCVA at 1 month				
LogMAR (mean ± SD)	-1.70 ± 0.96	-1.17 ± 1.17	-2.11 ± 0.48	0.16
Improvement of Postoperative BCVA at 3 months				
LogMAR (mean ± SD)	-1.69 ± 1.00	-1.10 ± 1.17	-2.14 ± 0.52	0.025
Improvement of Postoperative BCVA at 6 months				
LogMAR (mean ± SD)	-1.73 ± 0.97	-1.13 ± 1.12	-2.19 ± 0.52	0.038

*ILM* internal limiting membrane, *BCVA* best-corrected visual acuity, *logMAR* logarithm of the minimum angle of resolution, *SD* standard deviation

For cases with FAZ ischemia, the preoperative BCVA was 1.83 ± 0.99, and postoperative visual acuity at 3 months and 6 months were 0.14 ± 0.23 and 0.10 ± 0.23, respectively. In the ILM peeling group, the BCVA improvement was -1.10 ± 1.17 at 3 months and -1.13 ± 1.12 at 6 months. In the non-ILM peeling group, the BCVA improvement was -2.14 ± 0.52 at 3 months and -2.19 ± 0.52 at 6 months. In particular, the visual improvement at 3 months and 6 months in the ILM peeling group were significantly worse than that in the non-ILM peeling group (*P* = 0.025, *P* = 0.038).

However, among the cases without ischemia in the arcade, visual improvement at 3 and 6 months did not differ significantly between the ILM peeling group and the non-ILM peeling group at each point. (3 months postoperatively, *P* = 0.94; and 6 months postoperatively, *P* = 0.88).

In multivariable regression analysis, overall, the postoperative BCVA at 6 months was significantly associated with ischemia within the arcade (*P* = 0.024) and ILM peeling (*P* = 0.033), and the visual improvement at 6 months was significantly associated with the preoperative BCVA (P =  < 0.0001) and ILM peeling (*P* = 0.018). No significant association was found between the preoperative BCVA and postoperative BCVA at 6 months in cases with ischemia within the arcade (*P* = 0.32). In cases with ischemia in the arcade, the postoperative BCVA at 6 months was significantly associated with ILM peeling (*P* = 0.041), and the visual improvement at 6 months was significantly associated with the preoperative BCVA (P =  < 0.0001) and ILM peeling (*P* = 0.022). In cases without ischemia in the arcade, the visual improvement at 6 months was significantly associated with the preoperative BCVA (P =  < 0.008).

## Discussion

In this study, the VH was dense enough to warrant surgical intervention, resulting in significant preoperative visual impairment. After surgery, the dense hemorrhage was removed, and in cases where retinal function remained intact, near-normal visual acuity was achieved. Consequently, there was a substantial overall improvement in vision. Intraoperatively, certain surgeons consider whether to perform ILM peeling to prevent the development of postoperative ERM and reoperation for ERM in cases with VH due to RVO, because the necessity for reoperation can greatly diminish the overall satisfaction with the surgical outcome. In cases of PDR, which shares similarities with ischemic diseases, studies have reported better visual outcomes in patients with associated Vitreous Hemorrhage (VH) who undergo ILM peeling compared to those who do not [18]. By contrast, ILM peeling did not improve the visual acuity in some studies and even adversely enlarged the FAZ area, [19, 20] and delay the recovery time of vessel density and vessel length density [21, 22]. In this study, we found that ILM peeling prevents postoperative ERM and postoperative visual acuity improved in both ILM peeling group and non-ILM peeling group. However, the postoperative improvement of BCVA in eyes with ILM peeling was significantly worse than that in non-ILM peeling, and postoperative ERM did not affect postoperative visual acuity. Importantly, it was observed that patients with macular ischemia, as identified through intraoperative imaging, experienced less improvement in visual acuity in the ILM peeling group compared to the non-ILM peeling group. In the group of patients with ischemia within the arcade, the postoperative BCVA at 3 months and the improvement from preoperative BCVA at 6 months in the ILM peeling group were significantly worse than those in the non-ILM peeling group. Therefore, we further analyzed these cases using OCTA and FA for a more comprehensive examination. In the group with FAZ ischemia in OCTA or FA, the postoperative BCVA at 6 months in the ILM peeling group was significantly worse than those in the non-ILM peeling group. In the non-FAZ ischemia group, ILM peeling did not prevent visual acuity improvement, and visual acuity recovered equally well with and without ILM peeling. In the cases without macular ischemia, the visual acuity was not affected by ILM peeling, like that previously reported in PDR. However, ILM peeling did not improve visual acuity in patients considered to have macular ischemia. The macular ischemia may be more susceptible to the effects of ILM peeling depending on cases. As has been indicated in previous reports, [21] there is a possibility that ILM peeling could enlarge FAZ ischemia and potentially limit improvement in visual acuity. One potential explanation for the discrepancy with PDR is that, in PDR, ischemia is scattered, while in RVO, ischemia spans the whole area of occluded vasculature. Therefore, the influence on macular ischemia by ILM peeling should be further investigated and considered in clinical practice.

Another unexpected discovery was that the presence or absence of postoperative ERM did not significantly impact the visual acuity after surgery. In other words, because the postoperative ERM does not result in vision loss, surgeons have concerns, but ILM peeling may not be necessary for the patient.

This study shows that ILM peeling in BRVO may be acceptable in the absence of FAZ ischemia but should be avoided in the presence of FAZ ischemia. In patients presenting for the first time with vitreous hemorrhage, the presence of FAZ ischemia can only be determined postoperatively. Therefore, in this study, assuming that the presence or absence of ischemia will be determined during surgery, we decided to determine the presence or absence of ischemia using fundus photographs. As the result, ILM peeling should be avoided if ischemia is intraoperatively present in the arcade. If it is difficult to determine whether ischemia is present in the arcade intraoperatively, ILM peeling should be uniformly avoided because postoperative ERM did not affect visual acuity. In summary, ILM peeling should be avoided unless it is unequivocally confirmed that ischemia does not extend to the macula.

This study has several limitations, including its retrospective design and relatively small sample size. First, preoperative evaluation of macular ischemia can be challenging when patients first present for consultation after vision loss due to vitreous hemorrhage. This is because some patients may not become aware of the onset in cases where the contralateral eye is healthy, and when RVO spares the macula from ischemia. After VH occurs, the VH obscures the fundus view and an inability to perform OCTA. On the other hand, postoperative assessment of macular ischemia may not accurately represent the preoperative condition due to potential changes during surgery, including the effects of dye, the manipulation, retinal photodamage, and surgical time. Consequently, in this study, in addition to preoperative and postoperative evaluations, we also included an intraoperative assessment of macular and FAZ ischemia.

This method was employed as the probability of ischemic changes occurring during surgery is considered low, and should such changes arise, they are likely to be detected by the surgeon. It is believed that the intraoperative findings immediately after VH removal are very similar to the preoperative conditions, allowing these observations to serve as a proxy for preoperative assessment in patients who did not have a consultation before VH. The consensus between two graders was very high.

Second, the possibility exists that the ERM may have been present preoperatively. In this research, there were no prominent ERM folds detectable intraoperatively that could indicate the definite presence of ERM. However, the existence of subtle ERMs cannot be ruled out. Therefore, it may have been difficult to determine intraoperatively whether an ERM was present. As a solution to this issue, intraoperative OCT can be utilized to determine the presence or absence of ERM during surgery, making it possible to exclude ERMs that existed preoperatively. Third, the follow-up period in this study was 6 months, and it cannot be denied that the onset of ERM in BRVO may take a long time and the formation of postoperative ERM may have a little effect on visual acuity in this period. Meta-analyses of idiopathic ERM surgery indicated that ILM peeling shows improvement in visual outcomes over the long term, specifically after 12 months, compared to shorter-term follow-ups [23]. However, other meta-analyses revealed no significant difference in long-term outcomes between groups with and without ILM peeling [24]. Further studies are needed to increase the number of cases and follow-up period. This necessity arises because the group that underwent ILM peeling may exhibit different outcomes over extended observation periods compared to those observed during a six-month follow-up. Also, from the perspective of macular ischemia, the aforementioned potential factors, including the presence of ILM peeling, the effects of dye, the manipulation, retinal photodamage, and surgical time, may influence macular ischemia over the long term, suggesting that further longitudinal studies are needed to fully understand their impact. In the case of vitrectomy for macular hole, the impact of dyes on postoperative outcomes has been reported [25]. While the present study found no significant differences, it is essential to continue investigating this aspect in future research. Fourth, the degree of macular ischemia may influence postoperative visual acuity. Because of the limited number of patients who underwent OCTA or FA in this study, objective grading of ischemia, such as vascular area and stratification analysis, was not performed. As deep retinal vasculature in BRVO associated with visual function had been reported [22], the study including consideration of degrees of ischemia by OCTA should be further investigated. Fifth, in this study, the decision to perform ILM peeling was at the discretion of the surgeon, which could potentially influence the outcomes if the procedure was reserved for more severe cases. However, in the current research, we included the cases with simple VH, which is not indicative of severe conditions, thereby likely minimizing this impact.

## Conclusion

ILM peeling should not be performed in cases with macular ischemia as it may result in potential damage to the fovea and significantly less visual recovery. ILM peeling should only be considered when there is irrefutable evidence ensuring that the ischemia has not encroached upon the macular region. Furthermore, it should also be considered that ERM, which are prevented by performing ILM peeling, do not affect postoperative visual acuity.
